# Enabling Online Studies of Conceptual Relationships Between Medical Terms: Developing an Efficient Web Platform

**DOI:** 10.2196/medinform.3387

**Published:** 2014-10-07

**Authors:** Aaron Albin, Xiaonan Ji, Tara B Borlawsky, Zhan Ye, Simon Lin, Philip RO Payne, Kun Huang, Yang Xiang

**Affiliations:** ^1^Department of Biomedical InformaticsThe Ohio State UniversityColumbus, OHUnited States; ^2^Department of Computer Science and EngineeringThe Ohio State UniversityColumbus, OHUnited States; ^3^Biomedical Informatics Research CenterMarshfield Clinic Research FoundationMarshfield, WIUnited States; ^4^The Research InstituteNationwide Children's HospitalColumbus, OHUnited States

**Keywords:** UMLS, ontology, conceptual relationships

## Abstract

**Background:**

The Unified Medical Language System (UMLS) contains many important ontologies in which terms are connected by semantic relations. For many studies on the relationships between biomedical concepts, the use of transitively associated information from ontologies and the UMLS has been shown to be effective. Although there are a few tools and methods available for extracting transitive relationships from the UMLS, they usually have major restrictions on the length of transitive relations or on the number of data sources.

**Objective:**

Our goal was to design an efficient online platform that enables efficient studies on the conceptual relationships between any medical terms.

**Methods:**

To overcome the restrictions of available methods and to facilitate studies on the conceptual relationships between medical terms, we developed a Web platform, onGrid, that supports efficient transitive queries and conceptual relationship studies using the UMLS. This framework uses the latest technique in converting natural language queries into UMLS concepts, performs efficient transitive queries, and visualizes the result paths. It also dynamically builds a relationship matrix for two sets of input biomedical terms. We are thus able to perform effective studies on conceptual relationships between medical terms based on their relationship matrix.

**Results:**

The advantage of onGrid is that it can be applied to study any two sets of biomedical concept relations and the relations within one set of biomedical concepts. We use onGrid to study the disease-disease relationships in the Online Mendelian Inheritance in Man (OMIM). By crossvalidating our results with an external database, the Comparative Toxicogenomics Database (CTD), we demonstrated that onGrid is effective for the study of conceptual relationships between medical terms.

**Conclusions:**

onGrid is an efficient tool for querying the UMLS for transitive relations, studying the relationship between medical terms, and generating hypotheses.

## Introduction

Since Swanson’s discovery of the connection between fish oil and Raynaud’s syndrome via blood viscosity [1], transitive associations have been important sources of hypothesis generation in biomedical science. In Swanson’s paradigm, an association between concepts A and C may be possible if both are related to a third concept, B. A number of discoveries and hypotheses have been made under this model. For instance, Hristovski et al proposed literature-based discovery to search disease candidate genes [2], to investigate drug mechanisms [3], and to identify novel therapeutic approaches [4]. As another example, Petric et al used this model to study autism by literature mining and found the connection between autism and calcineurin [5]. With the Unified Medical Language System (UMLS), such transitive association studies are becoming more efficient and powerful in generating novel hypotheses.

In biomedical science, the UMLS [6] is the largest thesaurus widely used in various applications. It is a collection of more than 160 source vocabularies (version 2012AA). The UMLS consists of three parts: the Metathesaurus, Semantic network, and Specialist lexicon. The Metathesaurus is the main body of the UMLS and has over 2 million concepts, each with a concept unique identifier (CUI), and over 15 million links (associations) between pairs of CUIs. The UMLS Terminology Services (UTS), hosted by the National Library of Medicine, provides an online query tool for these concepts under its Metathesaurus browser. To make use of the rich information contained in the UMLS, the interactive biomedical discovery support system (BITOLA) developed by Hristovski et al [2,7] supports the input of UMLS CUIs, concept, semantic types, and chromosome locations, in searching for hypothetic relations such as disease candidate genes.

BITOLA is based on Swanson’s one transitive relationship model. It is quite natural to ask if multiple transitive relationships will generate more rich hypotheses. Wilkowski et al [8] showed that by extracting paths from a graph modeling the concept relations, it is possible to extend this one transitive relationship model to a multiple-transitive relationship model for novel hypothesis discovery. For the UMLS, if we consider each CUI as a vertex, and links connecting two CUIs as an edge, we obtain a graph modeling the UMLS. The transitively associated queries on the UMLS can be regarded as queries on the UMLS graph. In fact, a number of works [9-14] have successfully used multiple-transitive relationships in the UMLS to study the closeness between two medical concepts. However, these works have two major limitations.

First, similar to [8], they rely on ad-hoc path search algorithms, such as Depth-First Search (DFS), which limit their searching ability on very large graphs. This is because the running time of DFS or similar ad-hoc search algorithms is proportional to the size of the graph being searched. As a result, it is not efficient to perform a large number of searches on a large graph using these algorithms. Thus, these works put major limitations on their search ranges, such as within a very small number of data sources in the UMLS, or very short search paths (eg, no more than 5 concepts in a path in [11]), to reduce the search space and thus to reduce the search time. Second, they generally rely on the distance to determine the closeness between two concepts. Since the distance between two concepts is determined by the shortest transitive relationship(s) and does not take into account other non-shortest transitive relationships, a false shortest transitive relationship may nullify the whole hypothesis. Given this observation, we conclude that this is not as reliable as a measurement on a large collection of paths. In fact, the effective measurement of relationship between two concepts in [2] and [15] can be viewed as a measurement on a collection of very short paths.

To overcome the two limitations, we developed a k-neighborhood Decentralization Labeling Scheme (kDLS) to efficiently index the UMLS [16]. kDLS supports efficient path/distance queries on the whole UMLS, as well as a measurement on the closeness between any two UMLS concepts by a collection of paths found between them. Efficiently querying such a large graph is a significant challenge for the graph database community. In fact, even the very recent graph indexing scheme [17] does not demonstrate the ability to efficiently answer distance queries on graphs with similar size and density. kDLS utilizes the power-law property of the UMLS for designing the indexing algorithm and turns out to be very effective in indexing the UMLS for both answering graph queries and discovering knowledge. Explained briefly, the indexing algorithm of kDLS iteratively removes a high degree vertex from the UMLS graph and broadcasts its information to the remaining vertices in the *k* neighborhood of the removed vertex. When the indexing ends, each vertex has a list of records that is considered its label. By comparing the labels of two vertices, it is possible to find a collection of paths (including but not limited to shortest paths) between the two vertices. We have proven that kDLS is guaranteed to find at least one shortest path if the two vertices are within *k* hops on the UMLS graph. On average, the number of paths discovered by kDLS is much larger than by the DFS or the Breadth-First Search (BFS), as we have shown previously [16]. Subsequently, the measurement between two concepts is based on the number of paths discovered as well as their lengths. kDLS has demonstrated its power in medical concept coreference resolution in clinical text [18].

However, kDLS has several major disadvantages: (1) it does not take into account the semantic networks in the UMLS ontologies, (2) it does not accept natural language–based queries, and only accepts queries on UMLS CUIs, and (3) it is time costly and difficult to configure and use kDLS for one study, regardless of the size of the study. To address these disadvantages, we developed an efficient online conceptual study platform using Graph indexing, onGrid, to study the conceptual relationships between biomedical terms.

## Methods

### System Framework

The cost to load the kDLS index is a major limitation of kDLS. Typically, it requires more than 20GB of memory [16] and takes several hours to load the kDLS index into memory before it can be used to efficiently answer queries and output discovered results. To provide an efficient solution for studies on conceptual relationships between medical terms, we developed onGrid, an online conceptual study platform using Graph indexing. onGrid provides a user-friendly Web interface to accept natural language-based queries and convert the queries to index-based searching on the UMLS and is expected to support future graph index engines on the UMLS. In addition, we proposed a new indexing method for onGrid that takes into account the concept semantic types, and our study on conceptual disease relationships demonstrated the advantages of the proposed indexing method over the original kDLS indexing method.

**Figure 1:**
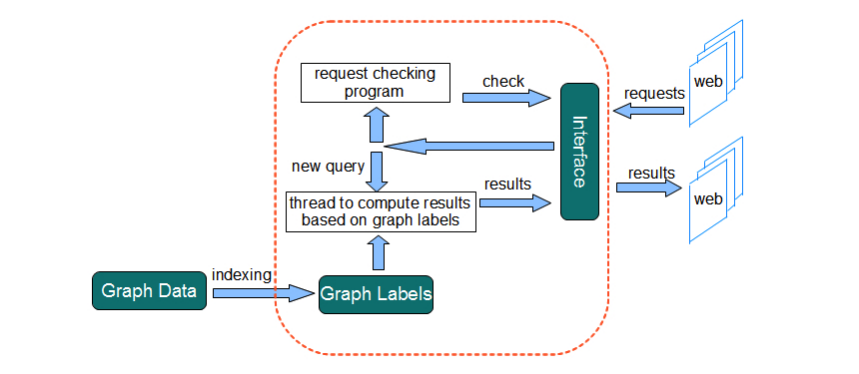
Flowchart of the onGrid framework.

The general framework of onGrid consists of two parts: the client side, which was implemented in JavaScript and PHP (Hypertext Preprocessor), and the server side, which was implemented in C++, a general purpose programming language. The client side receives query requests from users and transmits them to the server, which then executes the query requests and sends the results back to the client. This design pushes the light and fast pre-computation and post-computation tasks to the client side, which has limited resources, and the computing-intensive tasks to the server. To realize this, the server side program first loads the graph index into memory and iteratively checks for new requests from the client side. Once a new query request is received, the server side program dispatches a new thread to handle the request by using the loaded graph index. When the thread completes the request, it saves the results to be retrieved from the client. We use a MySQL database as the interface to facilitate the communication between the client side and the server side. All requests and results are posted to the database, which is regularly checked by both the server and client side programs. The flowchart of the system framework is illustrated in Figure 1.

### Web-Based Natural Language Processing

To enable natural language-based queries on the UMLS, we developed LDPMap [19], a layered dynamic programming approach that maps a biomedical concept to a UMLS concept. Since UMLS is very comprehensive, nearly all medical concepts can find their corresponding part in the UMLS. Our study shows that LDPMap is an effective tool for mapping a biomedical concept to a UMLS concept. In this work, we integrate LDPMap into onGrid such that biomedical terms in a query will be mapped to UMLS concepts before the query is executed. To avoid mapping errors, the system will automatically provide a list of mapped UMLS terms with CUIs in order of relevance for querying the relationship between two medical terms. Users can accurately select the terms for further querying.

### Network Visualization

Querying for relationships between two concepts returns a collection of paths between two query concepts. To provide users intuition on the path query results, onGrid visualizes the shortest paths among these paths. Visualizing all paths may not be feasible because the path query results often contain thousands of paths or more, which are hardly discernible considering the visual clutter. On visualizing the shortest paths between two vertices *u* and *v*, we organize all vertices that have the same distance to vertex *u* (or *v*) into a set *S*_*k*_ where *S*_*k*_ = ∪_p∈P’(u,v)_{*x*|*x*∈*p*, *distance*(*x*,*u*)=*k*} (*P*′(*u*,*v*) is the set of shortest paths among the collection of paths between *u* and *v*. All vertices in a set *S*_*k*_ will be visualized on a line perpendicular to the line connecting *u* and *v*. In this way, we are not only able to observe paths connecting two vertices but also observe shared vertices and edges among those paths.

### Concept Similarity Measurement

To measure the closeness between two concepts, onGrid takes into account the semantic type of each concept (vertex). UMLS (version 2012AA) has a total of 133 concept semantic types such as “Event”, “Disease or Syndrome”, etc. They are organized in a directed acyclic graph known as the UMLS concept semantic network. The semantic types closer to the root level are more abstract than those closer to the leaf level. Abstract semantic types are more likely to be related to a large number of concepts, and therefore we consider such relationships weak. To put more emphasis on concrete concepts in a path, the closeness between two concepts are measured by:

*R*(*u*,*v*)=∑_p∈P(u,v)_Π_x∈p_ *g*(*x*)

where *P*(*u*,*v*) is the collection of all paths between *u* and *v* discovered by kDLS, excluding paths with lengths equal to 1. *g*(*x*) is the semantic function on vertex (concept) *x*. In the onGrid implementation, we let *g*(*x*) = 1/*h* where *h* is the reverse topological level of vertex *x.* All leaves in the concept semantic network have reverse topological level 1. After removing all these leaves, all new leaves in the new network have reverse topological order 2. Iteratively applying this approach, we can determine a reverse topological level for all concept semantic types. When one concept has multiple semantic types, we assign the concept a semantic type closest to the leaves of the concept semantic network. Under this measurement, two concepts are likely to be close if there are many short and concrete paths between them.

## Results

### Transitive Relationship Queries and Visualization

**Figure 2:**
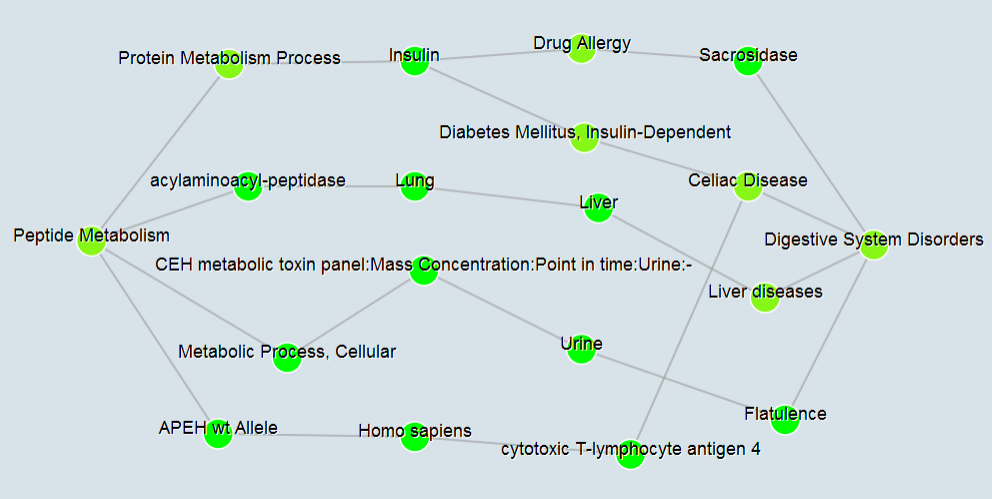
An illustration of visualized paths between two biomedical concepts.

onGrid supports both graph queries and conceptual relationship studies on UMLS data sources. For graph queries, it supports distance and shortest path queries on a conceptual network built upon UMLS data sources. To use this function, users can input a start biomedical concept (or CUI) and an end biomedical concept (or CUI), in which the system will output shortest paths visualized in a network structure. Figure 2 provides an illustration of such a network of structured paths between Peptide Metabolism (Semantic Type: Molecular Function) and Digestive System Disorders (Semantic Type: Disease or Syndrome). Users can choose to see an edge’s semantic type by moving their mouse to an edge (eg, Sacrosidase—“may_treat”—Digestive System Disorders), or simply selecting the option to show all of them.

In the current version, the basic settings of onGrid, including neighborhood search range, sink and source vertex handling, and semantic restrictions, follow our preliminary study [16], which demonstrates that this setting is cost-effective for knowledge discovery on the UMLS. In this setting, since k is configured to be 6, the system guarantees finding exact distances no more than 6 hops, or at least one shortest path no more than 6 hops, on the underlying graph built upon the selected UMLS data sources.

### Large Scale Relationship Matrix Generation

**Figure 3:**
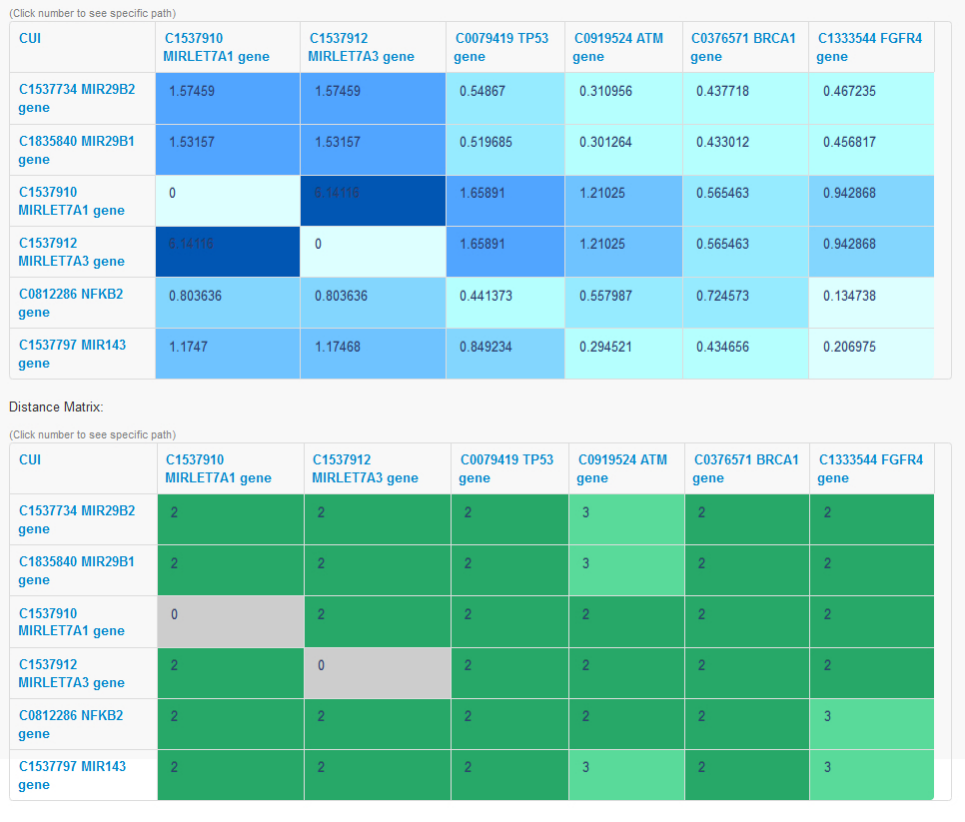
An example of relationship matrix and distance matrix generated by onGrid (in the relationship matrix, a higher number means a closer relationship).

In addition to the path queries, onGrid supports powerful conceptual relationship studies by allowing users to input two sets of biomedical concepts (or CUIs) and builds a distance heatmap/matrix as well as a relationship heatmap/matrix (as illustrated in Figure 3).

The distance heatmap provides a distance between every two concepts. However, distance alone may not be a good measurement for the relationships between medical concepts. Thus, onGrid provides the relationship heatmap using the concept relationship measurement function *R*(*u*,*v*) defined above, which extends the measurement in [16] by giving more weight to concrete paths, that is, paths with fewer abstract concepts. Similar to [16], paths with only one edge (ie, direct relations) are not counted in *R*(*u*,*v*) to avoid bias towards existing knowledge. Below we examine a large scale study on conceptual relationships between medical terms that uses the relationship matrix generated under this measurement. Finally, onGrid provides a very convenient feature for exploring these two matrices: If users are interested in any particular pair of CUIs, they can click the corresponding unit and onGrid will provide the result for the shortest path query on those two medical concepts.

onGrid also supports studies on large sets of medical concepts for users who wish to use this functionality due to the large amount of processing time required. onGrid supports these types of applications by allowing users to upload files, track their jobs, and download the results (a valid email address is required for these purposes).

### Validating onGrid by Studying Conceptual Relationships Between Diseases

onGrid can be applied to study the relations in a set of medical concepts or between two sets of medical concepts. To carry out the study, one can map these concepts to ontology terms in the UMLS using the natural language processing method described above and then generate a relationship matrix for these terms. In order to crossvalidate our results by an available source, we used onGrid to study the disease relationships in the Online Mendelian Inheritance in Man (OMIM) ontology dataset, which is a database collection of diseases with a genetic component. First, we use onGrid (on the full UMLS data source configuration) to generate a relationship matrix between diseases in OMIM and genes in the Human Genome Organization (HUGO). Then, given a threshold *δ*, we are able to convert the relationship matrix into a 0-1 relationship matrix. We construct weighted relations *T* over OMIM diseases by the number of genes shared by two diseases in the 0-1 relationship matrix. To crossvalidate our results, we build the same weighted disease relations *S* on the Comparative Toxicogenomics Database (CTD) [20]. We use fold enrichment to measure our results. The fold enrichment function is defined as f(*α*) = (|*S*′(*α*)|/|*S*′|)/(|*T*(*α*)|/|*T*|) where *S*′ = *S*∩*T*; *S*′(*α*) is the number of elements in *S* that are ranked in the top *α* percent of *T* according to the weight of disease pairs; *T*(*α*) is the number of elements in *T* that are ranked in the top *α* percent of *T*. It is quite intuitive that f(*α*) will be close to 1 if *T* is random, and if f(*α*) is much larger than 1, it suggests that *T* is statistically significant with respect to *S*.

Here we give a small hypothetical example to illustrate the above fold enrichment measurement. Let *T* = (<A,B> , <A,C> , <B,D> , <E,F> , <A,E> , <B,C> , <B,E> , <D,E> , <D,F> , <C,D>), which contains 10 pairs of diseases ranked in the descending order of their closeness. Let *S* = {<A,C>, <B,D>, <A,E>, <E,F>, <H,G>, <E,H>}, which contains six pairs of confirmed disease pairs. Then *S*′= *S*∩*T* = {<A,C>, <B,D>, <A,E>, <E,F>}, and *S*′(*α* = 20%) = {<A,C>}. Thus, the fold enrichment at *α* = 20% is f(*α* = 20%) = (|*S*′(*α*)|/|*S*′|)/(|*T*(α)|/|*T*|) = (1/4)/(2/10) = 1.25, and the maximum fold enrichment f(*α*) = 3.75 (when *α* = 40%).

**Figure 4:**
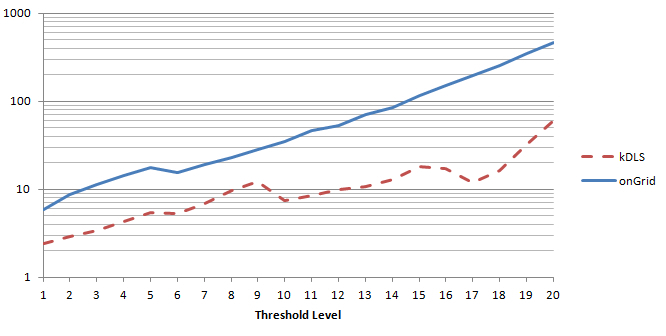
Maximum Fold Enrichment for both kDLS and onGrid.

**Figure 5:**
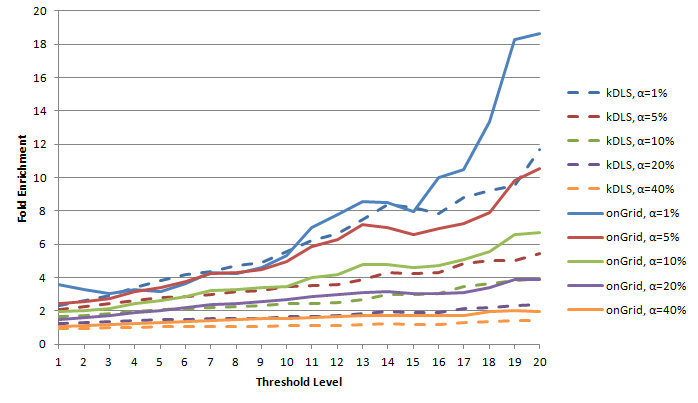
Fold Enrichment f(α) for both kDLS and onGrid.

The fold enrichment results of the OMIM disease relationships generated by onGrid with respect to CTD are provided in Figures 4 and 5. To understand the advantage of onGrid over kDLS, we also include the kDLS in the study.

From Figures 4 and 5, we can see that fold enrichment values are much larger than 1. They generally increase when the threshold *δ* increases. This is because when the threshold *δ* is high, only the disease pairs sharing the most genes (ie, most significant disease-disease pairs) are left for study. Thus, to avoid studying too few disease pairs, the thresholds in this study were set to an upper limit. We also noticed that these values get smaller when percentage *α* increases. This is understandable because according to the definition, when *α* increases, the difference between the numerator and denominator tends to get smaller, and f(*α*) = 1 when *α* = 100. These fold enrichment tests suggest that the disease pair results obtained by onGrid are statistically significant in the crossvalidation with an external dataset, CTD.

In addition, Figures 4 and 5 include corresponding results generated from the original kDLS algorithm (indicated by dashed lines). To ensure the results are comparable, the percentiles of relationships (ie, entries) for *δ* thresholds in the onGrid matrices were obtained and used to determine appropriate *δ* values for the kDLS matrices. Table 1 lists their respective *δ* values for each threshold level. onGrid tends to generate higher fold enrichment values for each respective *α*, suggesting that incorporating semantic types leads to more focused and correlated diseases and genes.

**Table 1:** Corresponding thresholds *δ* for kDLS and onGrid.

Threshold level	*δ* for onGrid	*δ* for kDLS
1	0.45	0.73
2	0.5	0.8
3	0.55	0.87
4	0.6	0.93
5	0.65	1
6	0.7	1.08
7	0.75	1.15
8	0.8	1.23
9	0.85	1.29
10	0.9	1.35
11	0.95	1.41
12	1	1.48
13	1.05	1.53
14	1.1	1.59
15	1.15	1.68
16	1.2	1.74
17	1.25	1.82
18	1.3	1.9
19	1.35	2
20	1.4	2.07

### Comparing onGrid and Comparative Toxicogenomics Database on Conceptual Disease Relationships

**Figure 6:**
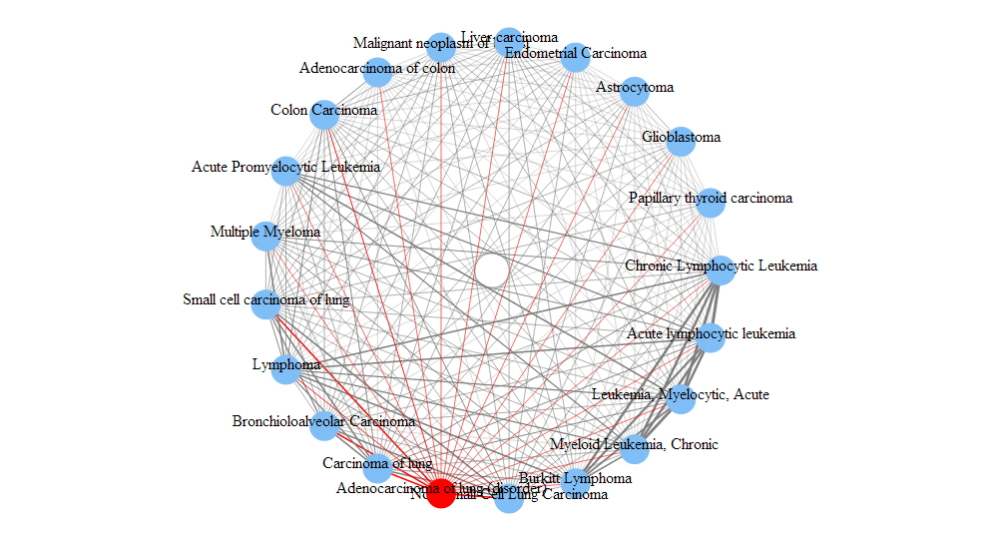
Top diseases related to adenocarcinoma of lung by onGrid (δ = 1.1).

**Figure 7:**
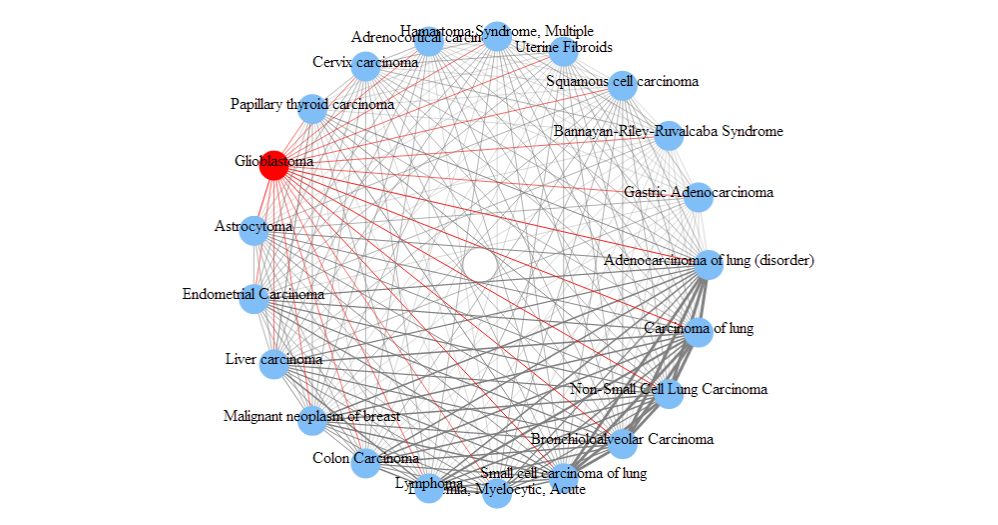
Top diseases related to glioblastoma by onGrid (δ = 1.1).

We are able to further study any interested diseases to observe other diseases most related to them. For demonstration purposes, we use adenocarcinoma of lung and glioblastoma in this study. The relationship between two diseases is measured by the number of genes shared between them. This measurement can be used to study the disease relationships in both onGrid results and in the CTD. According to the role of the threshold *δ*, one can infer that when *δ* decreases the differences among relationships (ie, edge thickness) blur, and when *δ* increases the differences among relationships become sharp, and at some point only the thickest edges will show. For conciseness in this paper, we show only the results under *δ* = 1.1 as a balanced result of the two effects. The top-ranked diseases related to the two diseases are presented in Figures 6 and 7 in circular arc graphs. The edges (relationships) connected to the studied diseases (adenocarcinoma of lung or glioblastoma) are shown in red, and other edges are shown in gray. An edge thickness is proportional to the normalized edge weight, which is obtained by categorizing the number of shared genes into 10 levels.

**Figure 8:**
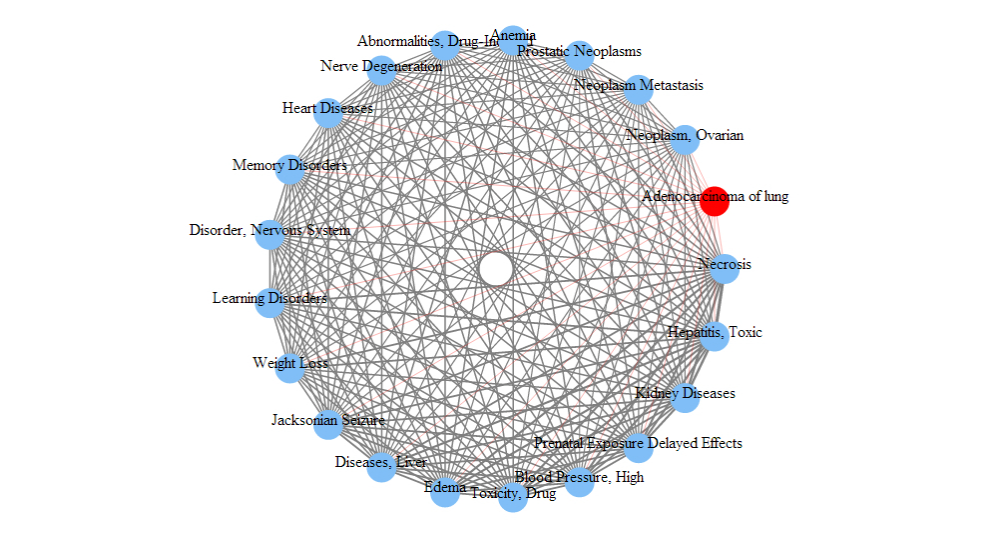
Top diseases related to adenocarcinoma of lung according to CTD.

**Figure 9:**
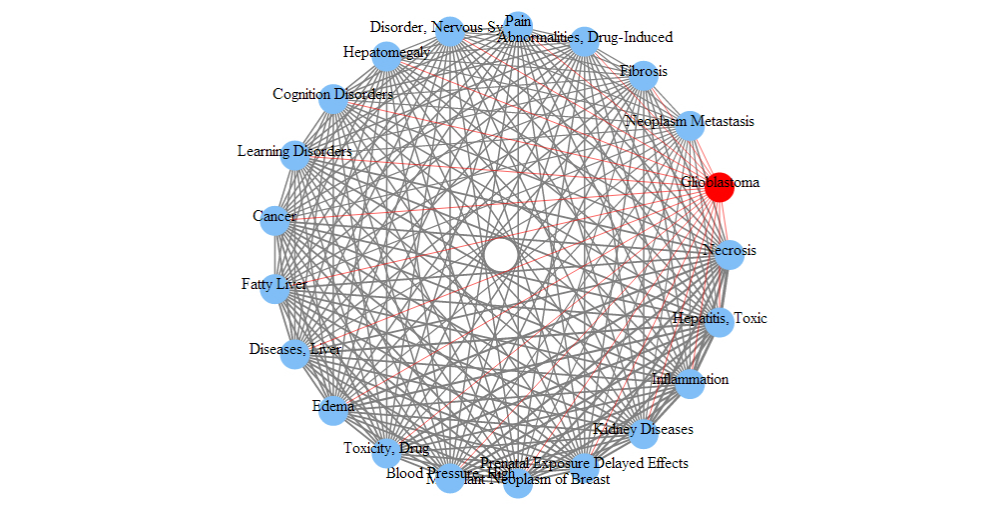
Top diseases related to glioblastoma according to CTD.

To demonstrate the advantage of onGrid, we also conducted the same analysis using CTD (Figures 8 and 9).

In Figures 6-9, we can see that the disease relationships generated by onGrid have a larger weight variation (visualized by the thickness of edges) compared to the disease relationships of CTD. Thus, it is easier to distinguish closeness between diseases in onGrid than CTD. In addition, the top-related diseases by onGrid (Figures 6 and 7) are mostly leukemia and carcinoma for adenocarcinoma of lung, and mostly carcinoma for glioblastoma. They are consistent with the disease mechanisms contained in the UMLS ontologies. Furthermore, we found that other independent studies partially confirm the results generated by onGrid. For example, the loss of heterozygosity on chromosome 3p was observed for both patients of small cell carcinoma of lung and patients of adenocarcinoma of lung [21], validating their relationships revealed by onGrid. As another example, lymphoma, a top-related disease to adenocarcinoma of lung by onGrid, was observed to have the same effect with adenocarcinoma of lung in the combined inactivation of oncogenes MYC and K-ras in a study using mouse models [22]. Similarly, we also found studies between glioblastoma and top diseases related to glioblastoma by onGrid. In contrast, the top-related diseases by CTD (Figures 8 and 9) are quite general, mostly reflecting the toxicology viewpoints of liver necrosis and kidney damage. These observations suggest that onGrid provides important information for studying the conceptual relationships between diseases.

## Discussion

### Studying Concept Relationships Using onGrid

Above we have shown the effectiveness of using onGrid for studying disease-disease relationships. These results can be used to assist other studies such as analyzing electronic health records. In addition, onGrid can be used for studying many conceptual relationships other than disease-disease or disease-gene relationships. We can use onGrid to study the relationships among many important biomedical concepts, including drugs, diseases, genes, side effects, etc. To perform these studies, we may use corresponding ontologies such as RxNorm (for drugs), International Classification of Diseases, 9th Revision, Clinical Modification (ICD-9-CM) (for diseases), OMIM (for diseases with a genetic component), Gene Ontology (GO) (for genes), and Medical Dictionary for Regulatory Activities (MedDRA) (for side effects). These studies can be used to assist many biomedical applications, such as identifying drug side effects and drug repurposing candidates. We can further leverage these studies with research on external datasets or ontologies.

### Limitations of the Conceptual Relationship Study Using Unified Medical Language System

Since UMLS is a collection of ontologies, it is essentially a body of knowledge. Although knowledge discovery on such data will produce transitive associations that may not have been noticed before, it will not produce knowledge that is out of the given ontological data. Consequently, the discovered relationships are likely to concentrate on well-studied concepts. In addition, since UMLS does not provide a weight on the concept relationships, it is not clear how important a relationship is. Thus, a transitive relationship on the UMLS may not be reliable. onGrid provides an advanced heuristic solution by considering both the discovered paths and semantic types. The crossvalidation demonstrates that the discovered results are statistically significant in aggregation. However, for one individual relationship between two concepts, it is difficult to further identify its statistical significance with the given resource in the UMLS. To complement this disadvantage, onGrid provides the path query function for two concepts and visualizes the discovered paths. Thus, domain experts are able to manually verify the validity of the transitive relationships between them. We expect that, in the future, by integrating information from external data sources, we will be able to perform efficient conceptual relationship studies that exceed the limitation of UMLS.

### Conclusions

onGrid provides an efficient Web-based platform to perform conceptual relationship studies using the UMLS. The current version of onGrid uses graph indexing with semantic relations as its server side index engine and can be easily upgraded in the future. onGrid can efficiently output shortest paths between two medical concepts as well as build relationship and distance heatmaps. The relationship heatmap enables researchers to quickly identify highly related medical concepts and directly check the transitive relation between any two concepts on the heatmap by clicking the corresponding unit. Our study on the conceptual relationships between OMIM diseases demonstrates the effectiveness of using onGrid in studying medical concept relations. We expect onGrid will be used for many applications to assist conceptual relationship studies in the biomedical field.

## Abbreviations

BFS: breadth-first search
BITOLA: biomedical discovery support system
CTD: Comparative Toxicogenomics Database
CUI: concept unique identifier
DFS: depth-first search
GO: Gene Ontology
HUGO: Human Genome Organization
ICD-9-CM: International Classification of Diseases, 9th Revision, Clinical Modification
kDLS: K-neighborhood Decentralization Labeling Scheme
LDPMap: Layered Dynamic Programming Map
MedDRA: Medical Dictionary for Regulatory Activities
OMIM: Online Mendelian Inheritance in Man
onGrid: Online conceptual study platform using Graph indexing
UMLS: Unified Medical Language System
UTS: UMLS Terminology Services
